# Guideline for reporting systematic reviews of outcome measurement instruments (OMIs): PRISMA-COSMIN for OMIs 2024

**DOI:** 10.1186/s12955-024-02256-9

**Published:** 2024-07-09

**Authors:** Ellen B. M. Elsman, Lidwine B. Mokkink, Caroline B. Terwee, Dorcas Beaton, Joel J. Gagnier, Andrea C. Tricco, Ami Baba, Nancy J. Butcher, Maureen Smith, Catherine Hofstetter, Olalekan Lee Aiyegbusi, Anna Berardi, Julie Farmer, Kirstie L. Haywood, Karolin R. Krause, Sarah Markham, Evan Mayo-Wilson, Ava Mehdipour, Juanna Ricketts, Peter Szatmari, Zahi Touma, David Moher, Martin Offringa

**Affiliations:** 1 Child Health Evaluative Sciences, The Hospital for Sick Children Research Institute, Toronto, ON Canada; 2 Department of Epidemiology and Data Science, Amsterdam UMC, Vrije Universiteit Amsterdam, Amsterdam Public Health Research Institute, Amsterdam, Netherlands; 3 Institute of Work and Health, Toronto, ON Canada; 4 Department of Epidemiology & Biostatistics and Department of Surgery, Western University, London, ON Canada; 5 Li Ka Shing Knowledge Institute, St. Michael’s Hospital, Unity Health Toronto, Toronto, ON Canada; 6 Epidemiology Division and Institute of Health Policy, Management, and Evaluation, Dalla Lana School of Public Health, University of Toronto, Toronto, ON Canada; 7 Queen’s Collaboration for Health Care Quality Joanna Briggs Institute Centre of Excellence, Queen’s University, Kingston, Canada; 8 Department of Psychiatry, University of Toronto, Toronto, ON Canada; 9 Cochrane Consumer Network, London, UK; 10 OMERACT Patient Research Partner, Toronto, ON Canada; 11 Centre for Patient Reported Outcomes Research, Institute of Applied Health Research, University of Birmingham, Birmingham, UK; 12 Department of Human Neurosciences, Sapienza University of Rome, Rome, Italy; 13 IRCCS NEUROMED, Pozzilli, Isernia Italy; 14 Faculty of Dentistry, University of Toronto, Toronto, ON Canada; 15 Warwick Research in Nursing, Division of Health Sciences, Warwick Medical School, University of Warwick, Gibbet Hill, Coventry, UK; 16 Cundill Centre for Child and Youth Depression, Centre for Addiction and Mental Health, Hospital for Sick Children, University of Toronto, Toronto, ON Canada; 17 Department of Biostatistics & Health Informatics, Institute of Psychiatry Psychology & Neuroscience (IoPPN), King’s College London, London, UK; 18 Department of Epidemiology, UNC Gillings School of Global Public Health, 2101C McGavran-Greenberg Hall Chapel Hill, Chapel Hill, NC 27599 USA; 19 School of Rehabilitation Science, Faculty of Health Sciences, McMaster University, Hamilton, ON Canada; 20 Patient Partner, Halifax, NS Canada; 21 Division of Rheumatology, Department of Medicine, Schroeder Arthritis Institute, Krembil Research Institute, Toronto Western Hospital, University of Toronto, Toronto, ON Canada; 22 Centre for Journalology, Clinical Epidemiology Program, Ottawa Hospital Research Institute, Ottawa, ON Canada; 23 Institute of Health Policy, Management and Evaluation, University of Toronto, Toronto, ON Canada

**Keywords:** Systematic reviews, Outcome measurement instrument, Reporting guideline, Measurement properties, PRISMA, COSMIN

## Abstract

**Purpose:**

Although comprehensive and widespread guidelines on how to conduct systematic reviews of outcome measurement instruments (OMIs) exist, for example from the COSMIN (COnsensus-based Standards for the selection of health Measurement INstruments) initiative, key information is often missing in published reports. This article describes the development of an extension of the Preferred Reporting Items for Systematic Reviews and Meta-Analyses (PRISMA) 2020 guideline: PRISMA-COSMIN for OMIs 2024.

**Methods:**

The development process followed the Enhancing the QUAlity and Transparency Of health Research (EQUATOR) guidelines and included a literature search, expert consultations, a Delphi study, a hybrid workgroup meeting, pilot testing, and an end-of-project meeting, with integrated patient/public involvement.

**Results:**

From the literature and expert consultation, 49 potentially relevant reporting items were identified. Round 1 of the Delphi study was completed by 103 panelists, whereas round 2 and 3 were completed by 78 panelists. After 3 rounds, agreement (≥ 67%) on inclusion and wording was reached for 44 items. Eleven items without consensus for inclusion and/or wording were discussed at a workgroup meeting attended by 24 participants. Agreement was reached for the inclusion and wording of 10 items, and the deletion of 1 item. Pilot testing with 65 authors of OMI systematic reviews further improved the guideline through minor changes in wording and structure, finalized during the end-of-project meeting. The final checklist to facilitate the reporting of full systematic review reports contains 54 (sub)items addressing the review’s title, abstract, plain language summary, open science, introduction, methods, results, and discussion. Thirteen items pertaining to the title and abstract are also included in a separate abstract checklist, guiding authors in reporting for example conference abstracts.

**Conclusion:**

PRISMA-COSMIN for OMIs 2024 consists of two checklists (full reports; abstracts), their corresponding explanation and elaboration documents detailing the rationale and examples for each item, and a data flow diagram. PRISMA-COSMIN for OMIs 2024 can improve the reporting of systematic reviews of OMIs, fostering their reproducibility and allowing end-users to appraise the quality of OMIs and select the most appropriate OMI for a specific application.

**Note:**

In order to encourage its wide dissemination this article is freely accessible on the web sites of the journals: Health and Quality of Life Outcomes; Journal of Clinical Epidemiology; Journal of Patient-Reported Outcomes; Quality of Life Research.

**Supplementary Information:**

The online version contains supplementary material available at 10.1186/s12955-024-02256-9.

## Plain language summary

Outcome measurement instruments are tools that measure certain aspects of health. When researchers want to know which tool is the best for their study, they do something called a systematic review. They gather all facts about these tools from the scientific literature, put them together, and then make a decision on which tool is the best for their research. The problem is that too many systematic review reports about these tools are missing important information. This makes it hard for readers of these reviews to understand them clearly and to pick the best tool. This study tried to solve this problem by creating a new guideline, called “PRISMA-COSMIN for Outcome Measurement Instruments”. This guideline helps researchers to report their reviews on tools in a clear and thorough way. The study identified 54 things that should be reported in any review of tools, covering everything from the report's title to the discussion section. *PRISMA-COSMIN for Outcome Measurement Instruments* will make the reporting of these reviews of tools better, so people can understand them and choose the right tool for their needs.

## Introduction

An outcome measurement instrument (OMI) refers to the tool used to measure a health outcome domain. Different types of OMIs exist, such as questionnaires or patient-reported outcome measures (PROMs) and its variations, clinical rating scales, performance-based tests, laboratory tests, scores obtained through a physical examination or observations of an image, or responses to single questions [1, 2]. OMIs are used to monitor patients’ health status and evaluate treatments in research and clinical practice [3, 4]. Systematic reviews of OMIs synthesize data from primary studies on the OMIs’ measurement properties, feasibility, and interpretability to provide insight into the suitability of an OMI for a particular use [2]. Systematic reviews of OMIs are an important tool in the evidence-based selection of an OMI for research and/or clinical practice.

Several organizations have developed methodology for conducting systematic reviews of OMIs, including Outcome Measures in Rheumatology (OMERACT) [5], JBI (formerly Joanna Briggs Institute) [6], and the COnsensus-based Standards for the selection of health Measurement INstruments (COSMIN) initiative [2], the latter being the most widely used. Despite the availability of methodological guidance on the conduct of OMI systematic reviews, such reviews are often not reported completely [7–9]. For example, a recent study into the quality of 100 recent OMI systematic reviews shows that reporting is lacking on feasibility and interpretability aspects of OMIs, the process of data synthesis, raw data on measurement properties, and the number of independent reviewers involved in each of the steps of the review process (unpublished data). Incomplete reporting limits reproducibility and hinders the selection of the most suitable OMI for a specific application [10]. At present, a reporting guideline for systematic reviews of OMIs does not exist.

Reporting guidelines outline a minimum set of items to include in research reports, and their endorsement by journals has been shown to improve adherence, methodological transparency, and uptake of findings [11–13]. To improve the reporting of systematic reviews, the Preferred Reporting Items for Systematic reviews and Meta-Analyses (PRISMA) guideline was developed, containing a checklist, an explanation and elaboration (E&E) document, and flow diagrams [14]. Endorsement of PRISMA has resulted in improved quality of reporting and methodological quality of systematic reviews [15]. PRISMA has been updated in 2020 and is primarily focused on systematic reviews of interventions [16]. Although systematic reviews of OMIs share common elements with systematic reviews of interventions, there are also several differences: for example, in a systematic review of OMIs, multiple reviews (i.e., one review per measurement property) are often included [17], and effect measures and evidence synthesis methods are different in systematic reviews of OMIs. As such, some PRISMA 2020 items are not appropriate for systematic reviews of OMIs, other items need to be adapted, and some items that are important are not included.

There is thus a need for reporting guidance specifically for systematic reviews of OMIs [18], which might also help to reduce the ongoing publication of poor-quality reviews in the literature [7, 8]. This study therefore aimed to develop the PRISMA-COSMIN for OMIs 2024 guideline as a stand-alone extension of PRISMA 2020 [16]. New in reporting guideline development, this study also aimed to integrate patient/public involvement in the development of PRISMA-COSMIN for OMIs 2024, as patients/members of the public are ultimately impacted by the results of these systematic reviews.

## Methods

Details on integrating patient/public involvement in the development of PRISMA-COSMIN for OMIs 2024, our lessons learned and recommendations for future reporting guideline developers are outlined elsewhere [19]. Patient/public involvement has been reported according to the GRIPP2 short form reporting checklist in the current manuscript [20].

### Project launch and preparation

**Fig. 1:**
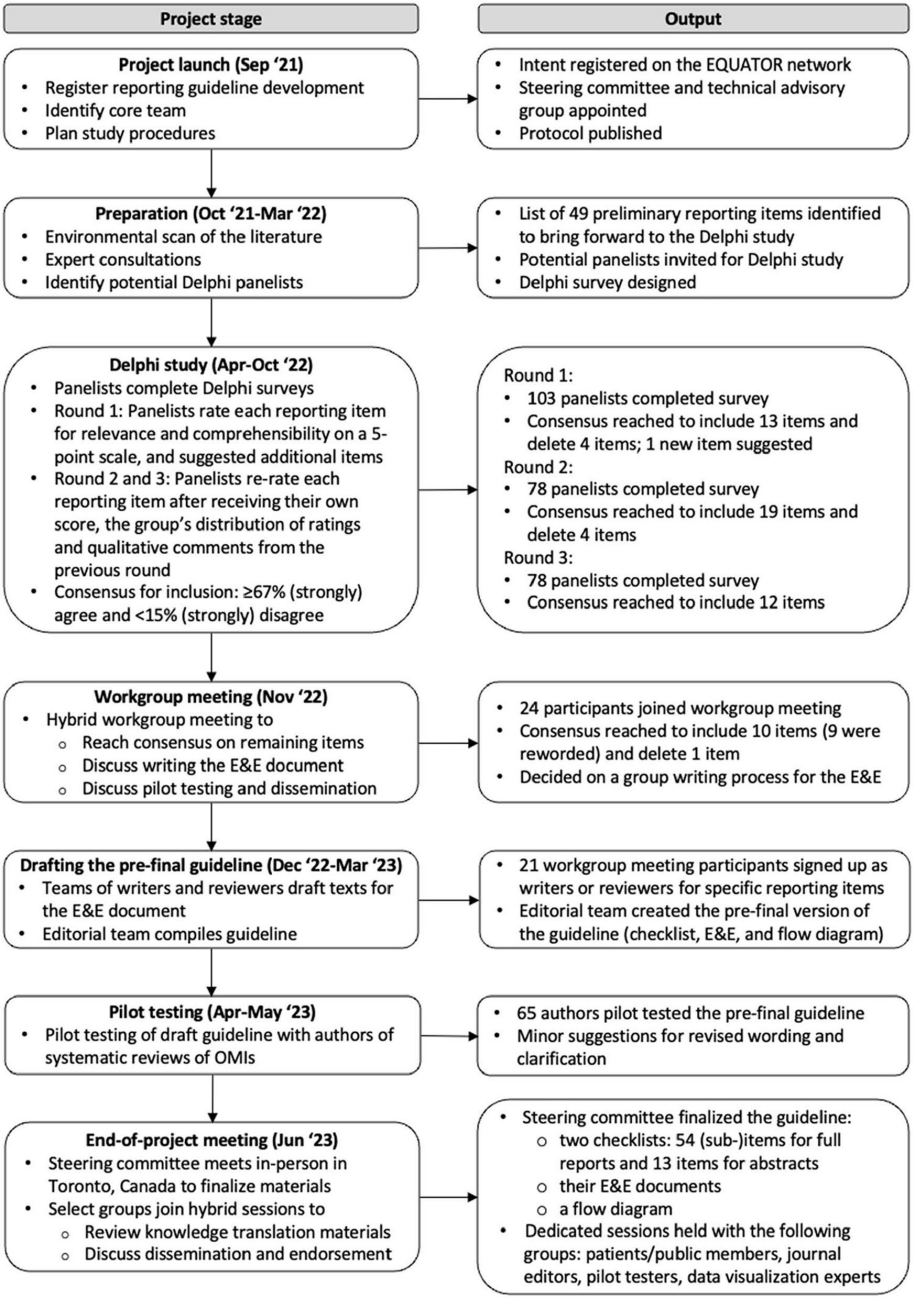
Development process of PRISMA-COSMIN for OMIs 2024. *E&E* explanation and elaboration; *EQUATOR* Enhancing the QUAlity and Transparency Of health Research; *OMI* outcome measurement instrument

We registered the development of PRISMA-COSMIN for OMIs 2024 on the Enhancing the QUAlity and Transparency Of health Research (EQUATOR) website [21] and the Open Science Framework [22]. Figure 1 shows the PRISMA-COSMIN for OMIs 2024 development process. A protocol was published previously [23] and Online Resource 1 states deviations from the protocol. The protocol details the project launch, preparation and PRISMA-COSMIN for OMIs 2024 item generation process. Briefly, a steering committee for project oversight, including a patient partner, and a technical advisory group for support and feedback were appointed (Online Resource 2 shows group membership). In the item generation process, we used PRISMA 2020 as the framework on which to modify, add, or delete items [16]. Potential items were identified by searching the literature for scientific articles and existing guidelines that describe potentially relevant reporting recommendations [2, 5, 6, 16, 24–51]. We applied this initial list of items to three different types of OMI systematic reviews: a systematic review of all available PROMs that measure a certain outcome domain in a certain population [52], a systematic review of one specific PROM [53], and a systematic review of a non-PROM (digital monitoring devices for oxygen saturation and respiratory rate) [54]. Application of the initial item list to these systematic reviews resulted in supporting, refuting, refining and supplementing the items. Findings were shared with the steering committee and technical advisory group, resulting in a list of preliminary items that were presented during the first round of the Delphi study [23].

### Delphi study

We conducted a 3-round international Delphi study between April and September 2022 using Research Electronic Data Capture (REDCap) [55]. The aim of the Delphi study was to obtain consensus on the inclusion and wording of items for PRISMA-COSMIN for OMIs 2024. We invited persons involved in the design, conduct, publication, and/or application of systematic reviews of OMIs as panelists. They were identified by the steering committee (researchers in the steering committee were not able to participate) and from other relevant Delphi studies [1, 40, 56–58]. Persons who co-authored at least three systematic reviews of OMIs, identified through the COSMIN database for systematic reviews [59], were also invited. Invitees could forward the invitation to other qualified colleagues. Besides the patient partner, we selected five patients/members of the public to join through newsletters and contact persons of relevant organizations [60–63]. Patients/members of the public attended a 90-min virtual onboarding session led by the patient partner and project lead with information about the purpose of the study, OMIs, systematic reviews, reporting guidelines, and the Delphi method. Support was offered throughout the process, if needed.

Registered panelists were invited for each round, irrespective of their responses to previous rounds. Each round was open for approximately four weeks, and weekly reminders were sent two weeks after the initial invitation. For each proposed item, panelists indicated whether it should be reported in a systematic review of OMIs, and whether the wording was clear. Both questions were scored on a five-point Likert scale: strongly disagree, disagree, neutral, agree, strongly agree. Panelists could also opt to select ‘not my expertise’; these responses were not included for calculating consensus. As decided a priori, consensus for inclusion was achieved when at least 67% of the panelists agreed or strongly agreed with a proposal [24, 56, 57, 64] and less than 15% disagreed or strongly disagreed [1, 58]. Panelists were encouraged to provide a rationale for their ratings and suggestions for improved wording.

In round 1, panelists also voted on original PRISMA 2020 items that were thought to have limited relevance for systematic reviews of OMIs. For these items, panelists indicated whether they were indeed *not* applicable, using the five-point Likert scale described above. In addition, panelists were asked to suggest new items not included in the list. Round 2 of the Delphi study included all round 1 items (except original PRISMA 2020 items that achieved consensus for inclusion and wording), as well as any new items that were suggested during round 1. If panelists made compelling arguments for the deletion of an item in round 1, this was brought forward in round 2, where panelists indicated whether they agreed with the deletion. Round 3 included items that did not reach consensus during rounds 1 or 2, or items with modified wording.

Following each round, frequencies of responses across all panelists and for each group (academia, patients/members of the public, other) were calculated. The project lead (EE) reviewed and summarized qualitative arguments to identify arguments against the overall trend in frequencies. The steering committee checked the summaries of qualitative arguments. A feedback report detailing frequencies and all anonymized qualitative comments was created and shared with panelists in each subsequent round. Each subsequent round also included the summary of qualitative arguments, the percentage consensus for inclusion and wording, and panelists’ own rating from the previous Delphi round.

### Workgroup meeting

We held a 3-h hybrid workgroup meeting in Toronto, Canada, and through Zoom in November 2022. This meeting was held to reach agreement on the inclusion and wording of items that had no consensus for inclusion after round 3 of the Delphi study, or for items for which the wording was revised. The steering committee selected participants with a variety of backgrounds from diverse geographic locations from the Delphi panelists who completed all three rounds; however, we did not use the specific responses of panelists in the Delphi study as a criterion for their selection to participate in the workgroup meeting. Additionally, certain members of the technical advisory group, knowledge users, and a limited number of editors were invited, irrespective of their participation in the Delphi study.

Ten days before the meeting, all attendees received an information package via email, including 1) an agenda, meeting details, and practical preparation steps for the meeting, 2) a full list of items detailing their changes over the Delphi rounds, specifying the items that needed discussion at the meeting, 3) the feedback report from Delphi round 3, and 4) short bio statements and photos from participants in the workgroup meeting. Attendees were asked to review the information prior to the meeting. A pre-meeting was held with patients/members of the public to go over the aims and materials for the workgroup meeting.

A facilitator presented each item selected to be discussed, providing a summary of Delphi round 3 results orally and visually on slides. For items that needed agreement on wording, the chair of the meeting summarized main points, and final wording was decided. Where consensus for inclusion was required, attendees voted on each item via a poll. Voting options were “include”, “exclude”, or “abstain”, and ≥ 70% include/exclude was needed for consensus [65], not taking the abstainers into account. The meeting was audio recorded and a notetaker documented the results of each poll, as well as the final wording of the items agreed upon.

### Developing the guideline

#### Drafting the pre-final guideline

After the workgroup meeting, we drafted the pre-final guideline, consisting of 1) the PRISMA-COSMIN for OMIs 2024 checklists (a checklist for full reports and a checklist for abstracts) with a glossary explaining technical terms used; 2) their respective explanation and elaboration (E&E) documents, including a rationale and detailed guidance for the reporting of each item; and 3) the PRISMA-COSMIN for OMIs 2024 flow diagram. We invited workgroup participants to contribute to drafting the E&E document by signing up for specific items in teams of two writers and two reviewers. We made explicit effort to align the wording and structure with PRISMA 2020 [16], as this is expected to facilitate the usability and uptake of PRISMA-COSMIN for OMIs 2024.

#### Pilot testing

Authors in the process of drafting or publishing their systematic review of OMIs, or who recently (2022/2023) published their review were eligible for pilot testing the pre-final guideline. Pilot testers were recruited through the network of the steering committee, by emailing corresponding authors of systematic reviews published in 2022/2023 included in the COSMIN database [59], and by emailing contact persons of ongoing or completed (but not yet published) systematic reviews of OMIs registered in PROSPERO between January 1, 2020, and January 1, 2023 [66]. Pilot testers received the pre-final guideline and were asked to apply it to their drafted, submitted or recently published systematic review of OMIs. Pilot testers provided feedback on the relevance and understandability of each item and its E&E text using a structured survey in REDCap [55]. Responses from pilot testers were reviewed and used to improve the guideline.

#### End-of-project meeting

We held a hybrid two-day end-of-project meeting in Toronto, Canada, and over Zoom in June 2023, with most members of the steering committee attending in-person. The main goals of the meeting were to finalize the guideline based on the feedback from the pilot testers and discuss its implementation, dissemination, and endorsement. We held hybrid sessions ranging from 60–90 min on Zoom with the following groups: patients/members of the public, journal editors, pilot testers, and data visualization/OMI systematic review experts. Two weeks before the meeting, attendees received an information package via email, including 1) the agenda, session aims, meeting details, and practical information, 2) the bios and photos from participants relevant to their session, and 3) any session-specific documents, if applicable. Attendees were asked to review the information ahead of the meeting.

## Results

### Delphi study

In total, 252 potential panelists were invited for the Delphi study, of which 81 registered (response rate 32%). Additionally, 38 persons registered through referral. One person withdrew before the start of the first Delphi round, resulting in 118 invited panelists for each round. Of these, 109 panelists responded to at least one round (Online Resource 3a); their characteristics are presented in Table 1. Round 1 was completed by 103 panelists, whereas rounds 2 and 3 were completed by 78 panelists.

**Table 1:** Characteristics of Delphi panelists and participants in the workgroup meeting

Self-reported characteristic	Delphi study (total *n* = 109); *n* (%)	Workgroup meeting (total *n* = 24); *n* (%)
Primary perspective		
Academia	94(86)	18(75)
Hospital	4(4)	1(4)
Industry	2(2)	1(4)
Government	1(1)	0(0)
Editor	1(1)	0(0)
Non-profit	1(1)	0(0)
Patient	4(4)	3(13)
Patient representative	1(1)	1(4)
Public member	1(1)	0(0)
Job title^a^		
PhD student	6(6)	2(10)
Research assistant	2(2)	1(5)
Postdoctoral research fellow	15(15)	2(10)
(Senior) researcher/research associate	16(16)	7(35)
(Senior) lecturer	7(7)	0(0)
(Assistant/associate) professor	35(34)	4(20)
Clinician/therapist (various)	12(12)	1(5)
Editor/reader/information specialist	5(5)	1(5)
Director/dean/chair	5(5)	2(10)
Country of workplace^b^		
UK	28(26)	3(13)
Canada	27(25)	16(67)
USA	15(14)	2(8)
Australia	12(11)	0(0)
Spain	9(8)	0(0)
Netherlands	5(5)	2(8)
Italy	3(3)	1(4)
Japan	2(2)	0(0)
Other^c^	8(7)	0(0)
Relevant group^a, d^		
OMI systematic review author	78(76)	12(60)
Systematic review author	66(64)	13(65)
OMI developer	62(60)	11(55)
Clinician	42(41)	4(20)
Core outcome set developer	32(31)	11(55)
Epidemiologist	21(20)	11(55)
Psychometrician/clinimetrician	18(17)	9(45)
Journal editor	17(17)	4(20)
Reporting guideline developer	12(12)	9(45)
Biostatistician	5(5)	0(0)
Highest level of education^a^		
Master’s degree	11(11)	3(15)
MD	10(10)	1(5)
PhD	56(54)	12(60)
MD/PhD	26(25)	4(20)
Expertise on systematic reviews of OMIs^a^		
High	57(55)	11(55)
Average	41(40)	8(40)
Low	5(5)	1(5)
Expertise on PRISMA^a^		
High	60(58)	12(60)
Average	36(35)	4(20)
Low	7(7)	4(20)
Expertise on COSMIN^a^		
High	37(36)	9(45)
Average	53(51)	8(40)
Low	13(13)	3(15)
Previously involved in research^e^		
As participant	5(58)	4(100)
As patient/public research partner	6(100)	4(100)
Previously involved in methodological research^e^		
As participant	2(33)	2(50)
As patient/public research partner	4(67)	3(75)
Previously involved in a Delphi study^e^	4(67)	3(75)

*COSMIN* COnsensus-based Standards for the selection of health Measurement Instruments; *OMI* outcome measurement instrument; *PRISMA* Preferred Reporting Items for Systematic reviews and Meta-Analyses

^a^Not asked to patients, patient representatives and public members; ^b^ Patients, patient representatives and public members were asked for their country of residence; ^c^ Other countries (all *n* = 1): Belgium, China, South Korea, Singapore, France, South Africa, New Zealand, Switzerland; ^d^ Panelists could select multiple responses; ^e^ Only asked to patients, patient representatives and public members

**Fig. 2:**
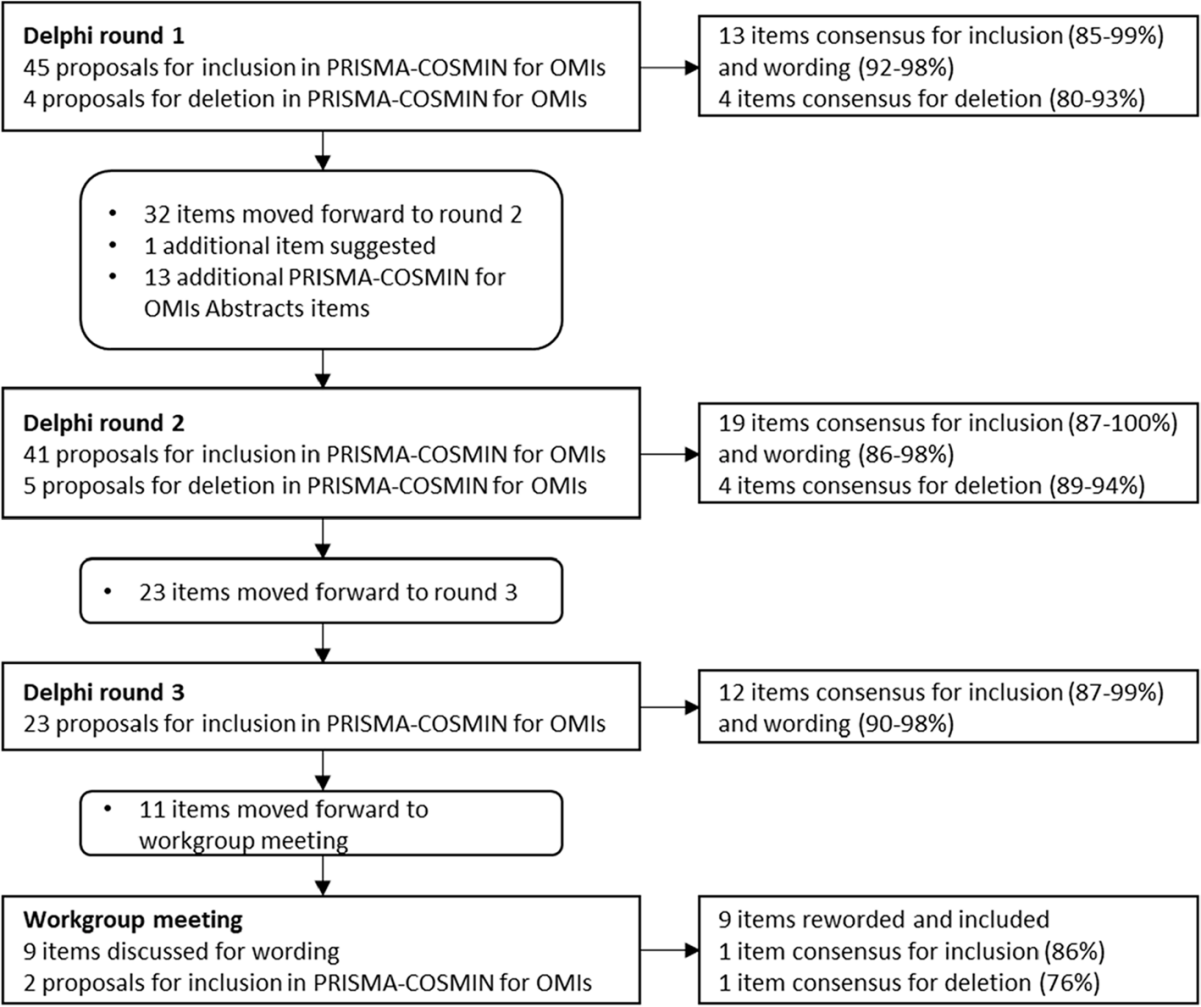
Proposals and consensus for items in each Delphi round and the workgroup meeting. *COSMIN* COnsensus-based Standards for the selection of health Measurement Instruments; *OMI* outcome measurement instrument; *PRISMA* Preferred Reporting Items for Systematic reviews and Meta-Analyses

In round 1, 49 potentially relevant items were proposed. Thirteen original PRISMA 2020 items reached consensus for inclusion and wording, whereas 4 original PRISMA 2020 items with limited relevance to systematic reviews of OMIs (related to data items, effect measures, and reporting biases) reached consensus for deletion (Fig. 2, Online Resource 4). Panelists made many qualitative arguments and suggestions for rewording. Wording was revised for all other items based on suggestions from panelists, and these items moved forward to round 2. For two items, related to the name and description of the OMI of interest and citing studies that appear to meet inclusion criteria but were excluded, panelists made compelling arguments for deletion in round 1. Panelists were asked to confirm deletion of these items in round 2, despite the high percentage of consensus for inclusion obtained in round 1.

While analyzing responses from round 1, we observed misunderstanding among panelists for the item pertaining to the abstract and for the items pertaining to the syntheses. Therefore, we extensively revised these items for round 2. Instead of one abstract item covering all elements, we added thirteen more specific abstract items, based on the PRISMA for Abstracts checklist [16, 67]. Three syntheses items were thought to be of limited relevance for systematic reviews of OMIs, and panelists were asked to confirm the deletion of these items in round 2. Based on suggestions for additional items, we drafted 1 new item pertaining to author contributions for consideration in round 2.

In round 2, 19 additional items reached consensus for inclusion and wording, whereas 4 items reached consensus for deletion (Fig. 2, Online Resource 4). Wording was revised for the other items based on suggestions of the panelists, and moved forward to round 3, despite having mostly high percentages of consensus for inclusion and wording.

In round 3, 12 additional items reached consensus for inclusion and wording (Fig. 2, Online Resource 4). Wording was slightly revised for 9 remaining items, although most of these items had high percentages of consensus for inclusion and wording. Consensus for inclusion was not reached for 2 items. These 11 items moved forward for discussion during the workgroup meeting.

Besides the confusion on the abstract item and syntheses items in round 1, panelists’ comments revolved around terminology for ‘studies’ and ‘reports’ as unit of analysis within these types of reviews. Within the context of measurement property evaluation, there is ongoing confusion about what constitutes a ‘study’. To avoid such confusion among review authors, we suggested to replace the PRISMA 2020 items that ask to report “the number of studies included in the review” by “the number of reports included in the review”. Ultimately, consensus on terminology was reached (see Table 4 for definitions of ‘study’, ‘report’, and ‘study report’) and the term “study reports” was used in those items.

Notably, patient/public involvement impacted the inclusion of reporting items pertaining to 1) feasibility and interpretability of the OMI, 2) recommendations on which OMI (not) to use, and 3) the plain language summary. Although other Delphi panelists saw little relevance for these items in the first Delphi round, patients/members of the public felt strongly about including these items. Their arguments ultimately persuaded other Delphi panelists to vote for inclusion of these items.

### Workgroup meeting

In total, 33 persons were invited to the hybrid workgroup meeting, of which 24 (72%) attended the meeting (16 through Zoom, 8 in-person). Attendants included nine steering committee members (one member was unable to attend), four members of the technical advisory group (all Delphi panelists), three knowledge users (two Delphi panelists), three patients/members of the public (all Delphi panelists), and five Delphi panelists (Online Resource 2). Their characteristics are presented in Table 1.

Through discussions, we reached agreement for wording for the 9 items that had their wording revised based on comments of panelists in Delphi round 3 (Fig. 2, Online Resource 4). Two items required voting on inclusion/deletion (one on citing reports that were excluded, one on author contributions). The first item reached 86% agreement for inclusion; the second item reached 76% agreement for deletion.

### Developing the guideline

All but three workgroup meeting participants contributed to drafting the E&E document for specific items. E&E text for each item was drafted by at least two writers and checked by at least 2 reviewers. Patients/members of the public signed up to be reviewers for reporting items that would benefit greatly from their input (e.g., items pertaining to the plain language summary, feasibility and interpretability of the OMI, and recommendations on which OMI (not) to use), as well as some other items, resulting in a clearer guideline. Select members of the steering committee made editorial edits for accuracy and consistency across items. A PRISMA-COSMIN for OMIs 2024 flow diagram was created.

We approached 515 potential pilot testers, of which 65 registered (response rate 13%). Additionally, 27 persons registered through referral, resulting in 92 registered pilot testers. These pilot testers were all in the process of drafting or publishing their systematic review, or recently published their review. Of these, 65 contributed to pilot testing by applying the guideline to their systematic review (Online Resource 3b); their characteristics are presented in Table 2. Pilot testers commented on the usability of the guideline and E&E document and made suggestions to improve clarity of the items and the E&E document.

**Table 2:** Characteristics of pilot testers

Self-reported characteristic	Pilot testers (total *n* = 65); n (%)
Job title	
(Post)graduate student	3(5)
PhD student	19(29)
Research assistant	6(9)
Postdoctoral research fellow	4(6)
(Senior) researcher/research associate	7(11)
(Senior) lecturer	8(12)
(Assistant) professor	11(17)
Clinician/therapist (various)	7(11)
Country of workplace	
Brazil	8(12)
UK	7(11)
Canada	6(9)
Germany	4(6)
Belgium	4(6)
Mexico	4(6)
Australia	3(5)
The Netherlands	3(5)
Italy	3(5)
Spain	3(5)
China	2(3)
Iran	2(3)
Switzerland	2(3)
Turkey	2(3)
United states	2(3)
Other^a^	10(15)
Highest level of education	
Bachelor’s degree	3(5)
Master’s degree	21(32)
MD	21(32)
PhD	5(8)
MD/PhD	9(14)
Other	6(9)
Participated in the Delphi study	
Yes	6(9)
No	59(91)
Use of guideline^b^	
As a checklist after drafting the review	55(93)
As a writing tool during drafting the rewiew	38(65)
As a peer-review tool for someone else’s review	14(24)
As a teaching tool	19(32)
Review used for pilot testing^c^	
Published	13(22)
Not yet published	46(78)
Role in review used for pilot testing^c^	
First author	45(76)
Co-author	8(14)
PI/senior author	6(10)

^a^Other countries (all *n* = 1): Greece, Ireland, Malaysia, Malta, Norway, Portugal, Saudi Arabia, Singapore, France, Sri Lanka; ^b^Only asked to participants who completed pilot testing (*n* = 59); participants could select multiple responses; ^c^Only asked to participants who completed pilot testing (*n* = 59)

Seven members of the steering committee met in-person for the two-day end-of-project meeting to finalize the guideline and E&E document based on the feedback of pilot testing, whereas two joined through Zoom (one was unable to attend). In addition, the following groups attended hybrid sessions: patients/members of the public (*n* = 5), journal editors (*n* = 7), pilot testers (*n* = 4), and data visualization/OMI systematic review experts (*n* = 6).

Feedback from pilot testing resulted in minor changes in wording and restructuring of the items, but not to changes in the content of the checklist. Most importantly, we changed the title of the section ‘other information’ to ‘open science’ and moved this section before the items on the introduction, consistent with the recently published CONSORT 2023 statement.

**Fig. 3:**
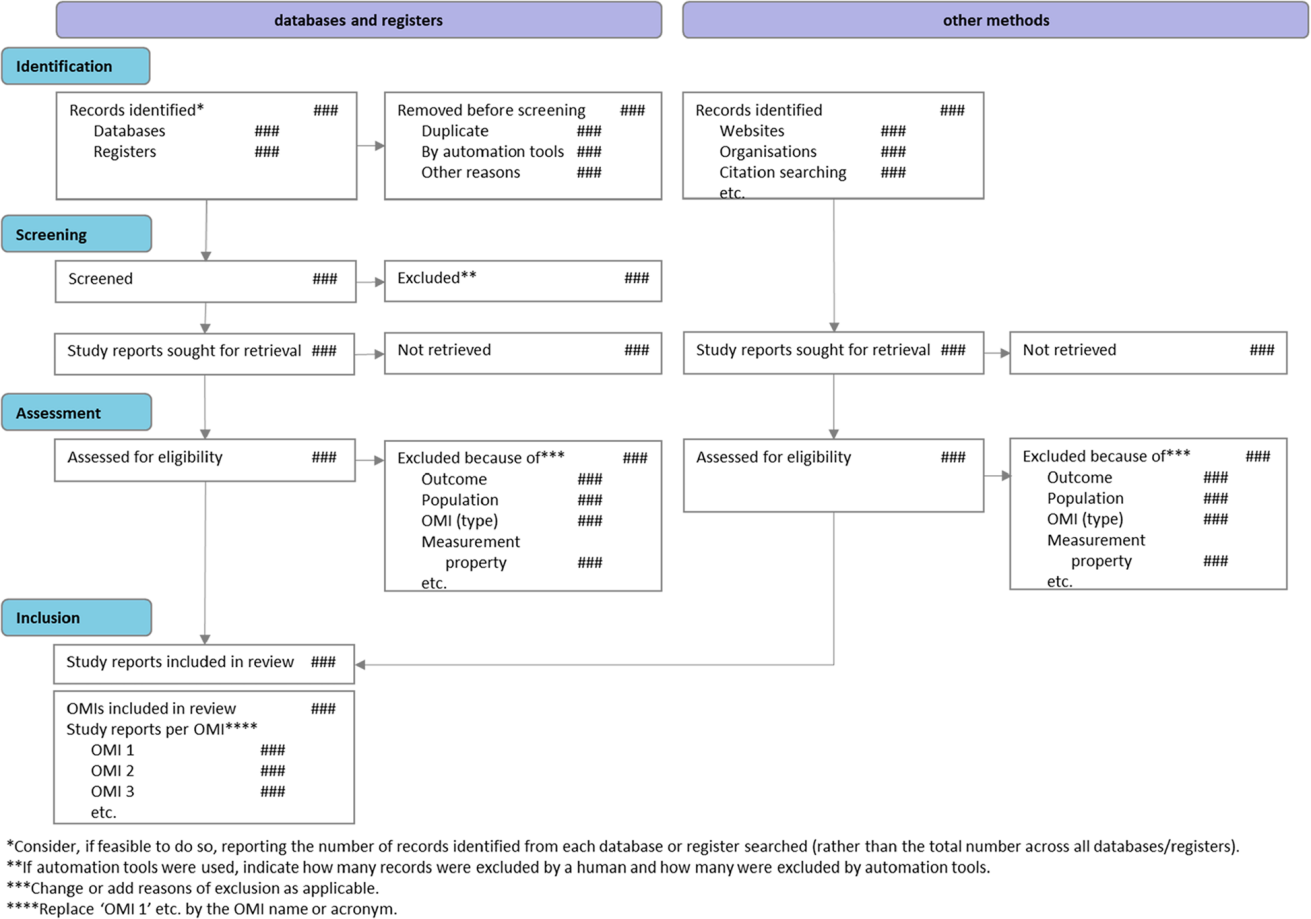
PRISMA-COSMIN for OMIs 2024 flow diagram

The PRISMA-COSMIN for OMIs 2024 guideline consists of a checklist for full systematic review reports with 54 (sub)items (Table 3), and a glossary of technical terms used (Table 4). The 13 items pertaining to the title and abstract are also included in a separate checklist that authors drafting e.g., conference abstracts could use. Their respective E&E documents (Online Resource 5 shows the E&E for full reports) contain a rationale for each item, essential and additional elements, and quoted examples from a published systematic review of OMIs. The PRISMA-COSMIN for OMIs 2024 flow diagram is shown in Fig. 3.

**Table 3:** PRISMA-COSMIN for OMIs 2024 checklist with Abstract items featured

Section and Topic	#	Checklist item^a^	Location
Title			
Title	1	Identify the report as a systematic review and include as applicable the following (in any order): outcome domain of interest, population of interest, name/type of OMIs of interest, and measurement properties of interest	
Abstract			
Open Science			
Funding^b^	2.2	Specify the primary source of funding for the review	
Registration	2.3	Provide the register name and registration number	
Background			
Objectives	2.4	Provide an explicit statement of the main objective(s) or question(s) the review addresses	
Methods			
Eligibility criteria	2.5	Specify the inclusion and exclusion criteria for the review	
Information sources	2.6	Specify the information sources (e.g., databases, registers) used to identify studies and the date when each was last searched	
Risk of bias	2.7	Specify the methods used to assess risk of bias in the included studies	
Measurement properties	2.8	Specify the methods used to rate the results of a measurement property	
Synthesis methods	2.9	Specify the methods used to present and synthesize results	
Results			
Included studies	2.10	Give the total number of included OMIs and study reports	
Synthesis of results	2.11	Present the syntheses of results of OMIs, indicating the certainty of the evidence	
Discussion			
Limitations of evidence	2.12	Provide a brief summary of the limitations of the evidence included in the review (e.g., study risk of bias, inconsistency, and imprecision)	
Interpretation	2.13	Provide a general interpretation of the results and important implications	
Plain Language Summary			
Plain language summary	3	If allowed by the journal, provide a plain language summary with background information and key findings	
Open Science			
Registration and protocol	4a	Provide registration information for the review, including register name and registration number, or state that the review was not registered	
	4b	Indicate where the review protocol can be accessed, or state that a protocol was not prepared	
	4c	Describe and explain any amendments to information provided at registration or in the protocol	
Support	5	Describe sources of financial or non-financial support for the review, and the role of the funders in the review	
Competing interests	6	Declare any competing interests of review authors	
Availability of data, code, and other materials	7	Report which of the following are publicly available and where they can be found: template data collection forms; data extracted from included studies; data used for all analyses; analytic code; any other materials used in the review	
Introduction			
Rationale	8	Describe the rationale for the review in the context of existing knowledge	
Objectives	9	Provide an explicit statement of the objective(s) or question(s) the review addresses and include as applicable the following (in any order): outcome domain of interest, population of interest, name/type of OMIs of interest, and measurement properties of interest	
Methods			
Followed guidelines	10	Specify, with references, the methodology and/or guidelines used to conduct the systematic review	
Eligibility criteria	11	Specify the inclusion and exclusion criteria for the review	
Information sources	12	Specify all databases, registers, preprint servers, websites, organizations, reference lists and other sources searched or consulted to identify studies. Specify the date when each source was last searched or consulted	
Search strategy	13	Present the full search strategies for all databases, registers, and websites, including any filters and limits used	
Selection process	14	Specify the methods used to decide whether a study met the inclusion criteria of the review, e.g., including how many reviewers screened each record and each report retrieved, whether they worked independently, and if applicable, details of automation tools/AI used in the process	
Data collection process	15	Specify the methods used to collect data from reports, e.g., including how many reviewers collected data from each report, whether they worked independently, any processes for obtaining or confirming data from study investigators, and if applicable, details of automation tools/AI used in the process	
Data items	16	List and define which data were extracted (e.g., characteristics of study populations and OMIs, measurement properties’ results, and aspects of feasibility and interpretability). Describe methods used to deal with any missing or unclear information	
Study risk of bias assessment	17	Specify the methods used to assess risk of bias in the included studies, e.g., including details of the tool(s) used, how many reviewers assessed each study and whether they worked independently, and if applicable, details of automation tools/AI used in the process	
Measurement properties	18	Specify the methods used to rate the results of a measurement property for each individual study and for the summarized or pooled results, e.g., including how many reviewers rated each study and whether they worked independently	
Synthesis methods	19a	Describe the processes used to decide which studies were eligible for each synthesis	
	19b	Describe any methods used to synthesize results	
	19c	If applicable, describe any methods used to explore possible causes of inconsistency among study results (e.g., subgroup analysis)	
	19d	If applicable, describe any sensitivity analyses conducted to assess robustness of the synthesized results	
Certainty assessment	20	Describe any methods used to assess certainty (or confidence) in the body of evidence	
Formulating recommendations	21	If appropriate, describe any methods used to formulate recommendations regarding the suitability of OMIs for a particular use	
Results			
Study selection	22a	Describe the results of the search and selection process, from the number of records identified in the search to the number of study reports included in the review, ideally using a flow diagram. If applicable, also report the final number of OMIs included and the number of study reports relevant to each OMI. [T]	
	22b	Cite study reports that might appear to meet the inclusion criteria, but which were excluded, and explain why they were excluded	
OMI characteristics	23a	Present characteristics of each included OMI, with appropriate references. [T]	
	23b	If applicable, present interpretability aspects for each included OMI. [T]	
	23c	If applicable, present feasibility aspects for each included OMI. [T]	
Study characteristics	24	Cite each included study report evaluating one or more measurement properties and present its characteristics. [T]	
Risk of bias in studies	25	Present assessments of risk of bias for each included study. [T]	
Results of individual studies	26	For all measurement properties, present for each study: (a) the reported result and (b) the rating against quality criteria, ideally using structured tables or plots. [T]	
Results of syntheses	27a	Present results of all syntheses conducted. For each measurement property of an OMI, present: (a) the summarized or pooled result and (b) the overall rating against quality criteria. [T]	
	27b	If applicable, present results of all investigations of possible causes of inconsistency among study results	
	27c	If applicable, present results of all sensitivity analyses conducted to assess the robustness of the synthesized results	
Certainty of evidence	28	Present assessments of certainty (or confidence) in the body of evidence for each measurement property of an OMI assessed. [T]	
Recommendations	29	If appropriate, make recommendations for suitable OMIs for a particular use	
Discussion			
Discussion	30a	Provide a general interpretation of the results in the context of other evidence	
	30b	Discuss any limitations of the evidence included in the review	
	30c	Discuss any limitations of the review processes used	
	30d	Discuss implications of the results for practice, policy, and future research	

^a^If an item is marked with [T], a template for data visualization is available. These templates can be downloaded from www.prisma-cosmin.ca. ^b^Item #2.1 in the PRISMA-COSMIN for OMIs 2024 Abstracts checklist refers to the title. Item #2.1 in the Abstracts checklist is identical to item #1 in the Full Report checklist

**Table 4:** Glossary of terms used in PRISMA-COSMIN for OMIs 2024

**Systematic review** A study design that uses explicit, systematic methods to collect data from primary studies, critically appraises the data, and synthesizes the findings descriptively or quantitatively in order to address a clearly formulated research question [65, 68, 69]. Typically, a systematic review includes a clearly stated objective, pre-defined eligibility criteria for primary studies, a systematic search that attempts to identify all studies that meet the eligibility criteria, risk of bias assessments of the included primary studies, and a systematic presentation and synthesis of findings of the included studies [65]. Systematic reviews can provide high quality evidence to guide decision making in healthcare, owing to the trustworthiness of the findings derived through systematic approaches that minimize bias [70]	
**Outcome domain** Refers to *what* is being measured (e.g., fatigue, physical function, blood glucose, pain) [1, 2]. Other terms include construct, concept, latent trait, factor, attribute	
**Outcome measurement instrument (OMI)** Refers to *how* the outcome is being measured, i.e., the OMI used to measure the outcome domain. Different types of OMIs exist such as questionnaires or patient-reported outcome measures (PROMs) and their variations, clinical rating scales, performance-based tests, laboratory tests, scores obtained through a physical examination or observations of an image, or responses to single questions [1, 2]. An OMI consists of a set of components and phases, i.e., ‘equipment’, ‘preparatory actions’, ‘collection of raw data’, ‘data processing and storage’, and ‘assignment of the score’ [57]. A specific type of OMIs is clinical outcome assessments (COAs) [71], which specifically focus on outcomes related to clinical conditions, often emphasizing the patient’s experience and perspective	
**Report** A document with information about a particular study or a particular OMI. It could be a journal article, preprint, conference abstract, study register entry, clinical study report, dissertation, unpublished manuscript, government report, or any other document providing relevant information such as a manual for an OMI or the PROM itself [68]. A study report is a document with information about a particular study like a journal article or a preprint	
**Record** The title and/or abstract of a report indexed in a database or website. Records that refer to the same report (such as the same journal article) are “duplicates” [68]	
**Study** The empirical investigation of a measurement property in a specific population, with a specific aim, design and analysis	
**Quality** The technical concept ‘quality’ is used to address three different aspects defined by COSMIN, OMERACT, and GRADE: 1) quality of the OMI refers to the measurement properties; 2) quality of the study refers to the risk of bias; and 3) quality of the evidence refers to the certainty assessment [2, 5, 72]	
**Measurement properties** The quality aspects of an OMI, referring to the validity, reliability, and responsiveness of the instrument’s score [64]. Each measurement property requires its own study design and statistical methods for evaluation. Different definitions for measurement properties are being used. COSMIN has a taxonomy with consensus-based definitions for measurement properties [64]. Another term for measurement properties is psychometric properties	
**Feasibility** The ease of application and the availability of an OMI, e.g., completion time, costs, licensing, length of an OMI, ease of administration, etc. [5, 26]. Feasibility is not a measurement property, but is important when selecting an OMI [2]	
**Interpretability** The degree to which one can assign meaning to scores or change in scores of an OMI in particular contexts (e.g., if a patient has a score of 80, what does this mean?) [64]. Norm scores, minimal important change and minimal important difference are also relevant concepts related to interpretability. Like feasibility, interpretability is not a measurement property, but is important to interpret the scores of an OMI and when selecting an OMI [2]	
**Measurement properties’ results** The findings of a study on a measurement property. Measurement properties’ results have different formats, depending on the measurement property. For example, reliability results might be the estimate of the intraclass correlation coefficient (ICC), or structural validity results might be the factor loadings of items to their respective scales and the percentage of variance explained	
**Measurement properties’ ratings** The comparison of measurement properties’ results against quality criteria, to give a judgement (i.e., rating) about the results. For example, the ICC of an OMI might be 0.75; this is the result. A quality criterion might prescribe that the ICC should be > 0.7. In this case the result (0.75) is thus rated to be sufficient	
**Risk of bias** Risk of bias refers to the potential that measurement properties’ results in primary studies systematically deviate from the truth due to methodological flaws in the design, conduct or analysis [68, 73]. Many tools have been developed to assess the risk of bias in primary studies. The COSMIN Risk of Bias checklist for PROMs was specifically developed to evaluate the risk of bias of primary studies on measurement properties [44]. It contains standards referring to design requirements and preferred statistical methods of primary studies on measurement properties, and is specifically intended for PROMs. The COSMIN Risk of Bias tool to assess the quality of studies on reliability or measurement error of OMIs can be used for any type of OMI [57]	
**Synthesis** Combining quantitative or qualitative results of two or more studies on the same measurement property and the same OMI. Results can be synthesized quantitatively or qualitatively. Meta-analysis is a statistical method to synthesize results. Although this can be done for some measurement properties (i.e., internal consistency, reliability, measurement error, construct validity, criterion validity, and responsiveness), it is not very common in systematic reviews of OMIs because the point estimates of the results are not used. Instead, the score obtained with an OMI is used. End-users therefore only need to know whether the result of a measurement property is sufficient or not. For some measurement properties it is not even possible to statistically synthesize the results by meta-analysis or pooling (i.e., content validity, structural validity, and cross-cultural validity/measurement invariance). In general, most often the robustness of the results is described (e.g., the found factor structure, the number of confirmed and unconfirmed hypotheses), or a range of the results is provided (e.g., the range of Cronbach’s alphas or ICCs)	
**Certainty (or confidence) assessment** Together with the synthesis, often an assessment of the certainty (or confidence) in the body of evidence is provided. Authors conduct such an assessment to reflect how certain (or confident) they are that the synthesized result is trustworthy. These assessments are often based on established criteria, which include the risk of bias, consistency of findings across studies, sample size, and directness of the result to the research question [2]. A common framework for the assessment of certainty (or confidence) is GRADE (Grading of Recommendations Assessment, Development, and Evaluation) [72]. A modified GRADE approach has been developed for communicating the certainty (or confidence) in systematic reviews of OMIs [2]	
**OMI recommendations** Systematic reviews of OMIs provide a comprehensive overview of the measurement properties of OMIs and support evidence-based recommendations for the selection of suitable OMIs for a particular use. Unlike systematic reviews of interventions, systematic reviews of OMIs often make recommendations about the suitability of OMIs for a particular use, although in some cases this might not be appropriate (e.g., if restricted by the funder). Making recommendations also facilitates much needed standardization in use of OMIs, although their quality and score interpretation might be context dependent. Making recommendations essentially involves conducting a synthesis at the level of the OMI, across different measurement properties, taking feasibility and interpretability into account as well. Various methods and tools for OMI recommendation exist (e.g., from COSMIN, OMERACT and others) [2, 74, 75]	

## Discussion

This paper outlines the development of PRISMA-COSMIN for OMIs 2024, including a Delphi study, workgroup meeting, pilot testing and an end-of-project meeting, and contains the checklist and E&E document for full reports. PRISMA-COSMIN for OMIs 2024 is intended to guide the reporting of systematic reviews of OMIs, in which at least one measurement property of at least one OMI is evaluated. These systematic reviews support decision making on the suitability of an OMI for a specific application. PRISMA-COSMIN for OMIs 2024 is not intended for reviews that only provide an overview (characteristics) of OMIs used, as these reviews are more scoping in nature. Systematic reviews of OMIs conducted with any methodology can use PRISMA-COSMIN for OMIs 2024; it does not apply specifically to systematic reviews conducted with the methodology or tools from the COSMIN initiative, although it is consistent with COSMIN guidance [2].

Similar to PRISMA 2020 [16], PRISMA-COSMIN for OMIs 2024 consists of two checklists (one for full reports and one for abstracts), their respective E&E documents, and a flow diagram. To develop PRISMA-COSMIN for OMIs 2024, we adapted PRISMA 2020 and made the following revisions to the checklist for full reports: 9 new items were added, 8 items were deleted because they were deemed not relevant for systematic reviews of OMIs, 24 items were modified, and 22 items kept as original. This checklist thus contains 54 (sub)items addressing the title, abstract, plain language summary, open science, introduction, methods, results, and discussion sections of a systematic review report. The 13 items pertaining to the title and abstract are also included in a separate Abstract checklist, accompanied by a separate E&E document that authors could use when drafting abstracts (e.g., conference abstracts).

The rigorous development process ensured that PRISMA-COSMIN for OMIs 2024 was informed by the knowledge of those who have expertise in OMIs and OMI systematic review methods, and patients/members of the public with lived experience. We were fortunate to include a good cross-section of stakeholders. Pilot testing with a large sample of authors of various OMI systematic reviews further improved PRISMA-COSMIN for OMIs 2024, confirming its broad applicability to different types and fields of OMI systematic reviews. We included patients/members of the public in the development process, as they are ultimately impacted by the results of systematic reviews of OMIs and the OMIs that are selected based on these reviews. Impact of patient/public involvement was evident, as four items were included that might have been disregarded, and their suggestions for rewording made the guideline clearer. As patient/public involvement in reporting guideline development is still in its infancy [76], we extensively evaluated this part of the process, reflected on lessons learned and provide recommendations for future reporting guideline developers elsewhere [19].

The field of evaluating OMIs is continuously evolving. For the development of PRISMA-COSMIN for OMIs 2024, we took PRISMA 2020 [16] as a guiding framework and used consensus methodology to modify, add, and delete reporting items based on the OMI literature and existing guidelines. The COSMIN guideline for systematic reviews of OMIs [2] was particularly important, as this currently is the most comprehensive and widely used guideline. Novel developments to evaluate OMIs, such as modern validity theory [77, 78] and qualitative research methods to investigate the impact of response processes and consequences of measurement [79, 80], might become increasingly important. Review authors who apply these methods are also able to use PRISMA-COSMIN for OMIs 2024 to guide their reporting. We will monitor the need for adaptations to the guideline should these methods be applied more frequently in OMI systematic reviews and require specific additional reporting items.

Despite the rigorous development process, we cannot be certain that we would have obtained exactly the same results if we would have done the process again, either with the same or with different participants. For example, in the Delphi study and workgroup meeting, we had relatively low representation of people from lower- and middle-income countries. This might have impacted our results, although representation in the pilot study was better. Another potential limitation is that we did not systematically search the literature to identify potential items in the preparation phase of the process. This was largely for pragmatic reasons, as we assumed that not much information on reporting recommendations for systematic reviews of OMIs would exist, as opposed to reporting guidance for primary studies on measurement properties [40]. Instead, we took PRISMA 2020 [16] as an evidence-informed and consensus-based framework and, based on our experiences with conducting, authoring, and reviewing systematic reviews of OMIs, we modified, added or deleted items. By applying the initial item list to three high-quality OMI systematic reviews we were able to confirm the relevance of items. The Delphi study and pilot testing with large and diverse samples validated these decisions. Moreover, our definition of consensus (67%) is somewhat arbitrary, although it has been used in other Delphi studies [24, 57, 64]. However, we ultimately reached at least 80% agreement on inclusion and wording in the Delphi study, so even if we had used a higher cut-off, this would not have changed our results.

Complete and transparent reporting of systematic reviews of OMIs is essential to foster reproducibility of systematic reviews and allow end-users to select the most appropriate OMI for a specific application. We hope that PRISMA-COSMIN for OMIs 2024 will improve the reporting of systematic reviews of OMIs as well as the quality of such reviews [7, 8]. PRISMA-COSMIN for OMIs 2024 will be published on the websites of the EQUATOR network, PRISMA, COSMIN, and www.prisma-cosmin.ca. To promote its uptake, a social media campaign to increase awareness, a short video (2–3 min) explaining the resources available to guide reporting systematic reviews of OMIs, and 1-page tip sheets outlining how to report each item will be created, in addition to patient-targeted materials. Furthermore, we are considering an automated e-mail system, whereby authors who register their OMI systematic review in PROSPERO [66] receive PRISMA-COSMIN for OMIs 2024. We will monitor the need for updating PRISMA-COSMIN for OMIs 2024, to reflect changes in best practice health research reporting and to stay consistent with PRISMA terminology.

### Supplementary Information

Supplementary Material 1.

Supplementary Material 2.

Supplementary Material 3.

Supplementary Material 4.

Supplementary Material 5.

## Data Availability

All data supporting the findings of this study are available within the paper and its Supplementary Information. Individual, anonymized responses from panelists or pilot testers are available from the corresponding author upon reasonable request.

## Abbreviations

COSMIN: COnsensus-based Standards for the selection of health Measurement Instruments
E&E: Explanation and elaboration
EQUATOR: Enhancing the QUAlity and Transparency Of health Research
JBI: Joanna Briggs Institute
OMERACT: Outcome Measures in Rheumatology (OMERACT)
OMI: Outcome measurement instrument
PRISMA: Preferred Reporting Items for Systematic Reviews and Meta-Analyses
PROM: Patient-Reported Outcome Measure
REDCap: Research Electronic Data Capture
